# Rupture of distal anterior cerebral artery aneurysm presenting only subdural hemorrhage without subarachnoid hemorrhage: a case report

**DOI:** 10.1186/s40064-016-1727-2

**Published:** 2016-01-26

**Authors:** Tae-Wook Song, Sung-Hyun Kim, Seung-Hoon Jung, Tae-Sun Kim, Sung-Pil Joo

**Affiliations:** Department of Neurosurgery, Chonnam National University Hospital and Medical School, 42, Jebong-ro, Donggu, Gwangju, 501-757 Republic of Korea

**Keywords:** Spontaneous subdural hematoma, Ruptured aneurysm, Subarachnoid hemorrhage

## Abstract

Intracranial aneurysm rupture usually manifests with subarachnoid hemorrhage, often combined with intracerebral hemorrhage with intraventricular hemorrhage extension. In rare cases, however, these aneurysms present only as subdural hematomas. Recently, we treated a 48-years-old female patient who presented only with subdural hematoma. Interestingly, she did not have a history of trauma. Computed tomography angiography and digital subtraction angiography revealed a 5 × 3 mm sized aneurysm at the A3–A4 junction of the left anterior cerebral artery. On admission, emergency operation (clipping and hematoma evacuation) was performed to protect against re-bleeding. Along with postoperative intensive care, the patient returned to normal daily life with only a mild headache. Given that patients may present with atraumatic acute subdural hematoma, the clinician must bear in mind the possibility of intracranial vascular pathology and obtain angiographic scans to evaluate for any underlying conditions to prevent patient deaths.

## Introduction

Aneurysm rupture usually presents as a subarachnoid hemorrhage (SAH), often in combination with intracerebral hemorrhage (ICH) and extension into the ventricle (intraventricular hemorrhage, IVH) (Barton and Tudor 1982; Mansour et al. 2014). In rare cases, acute subdural hematoma (aSDH) combined with SAH due to aneurysm rupture has been reported (Biesbroek et al. 2012, 2013; Kocak et al. 2009; Marbacher et al. 2010; Nozar et al. 2002), with only a few cases reported since the first case was reported in 1855 (Kocak et al. 2009; Marbacher et al. 2010). According to the literature, the incidence of aSDH due to aneurysmal rupture varies from 0.5 to 22 % (Barton and Tudor 1982; Biesbroek et al. 2012; Marbacher et al. 2010). The majority of patients with aSDH have sustained trauma, directly responsible for injury to bridging or cortical veins that ultimately leads to the subdural collection of blood with SAH combined with aSDH a have worse prognosis than patients without aSDH (Biesbroek et al. 2013). Uncommonly, other causes of spontaneous aSDH include arteriovenous malformations, cocaine abuse, neoplasms, and dural arteriovenous fistulars, to name a few (Alves and Gomes 2000).

Here we report an interesting case of spontaneous aSDH without a history of trauma due to aneurysmal rupture at the A3–A4 junction of the anterior cerebral artery (ACA). In addition, we elaborate on possible mechanisms of spontaneous aSDH without head trauma.

### Case report

**Fig. 1:**
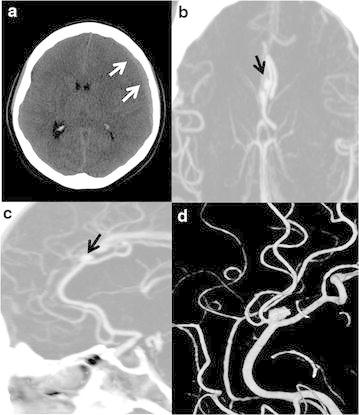
**a** Non-contrast brain CT shows acute subdural hematoma in the left fronto-tempo-parietal area (*white arrow*). The *midline* is shifted about 6 mm to the right. **b**, **c** Brain CTA reveals an A3–A4 junction aneurysm (*black arrow*). **d** 3D-DSA reveals an aneurysm at the A3–A4 junction

**Fig. 2:**
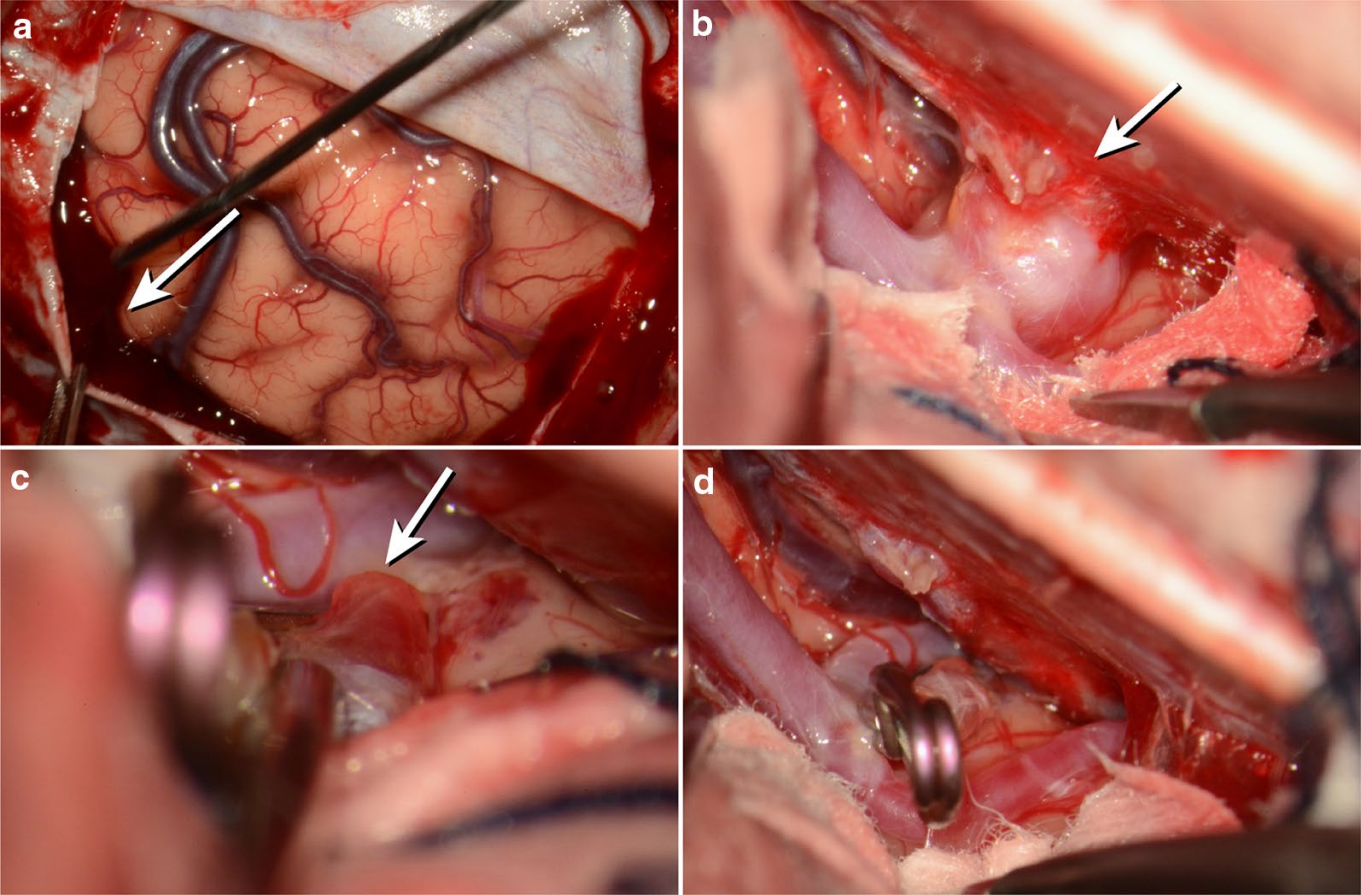
**a** Hematoma in the subdural space (*arrow*). **b** Aneurysm adhering to the falx (*arrow*). **c** Ruptured bled (*arrow*). **d** Complete clipping state

**Fig. 3:**
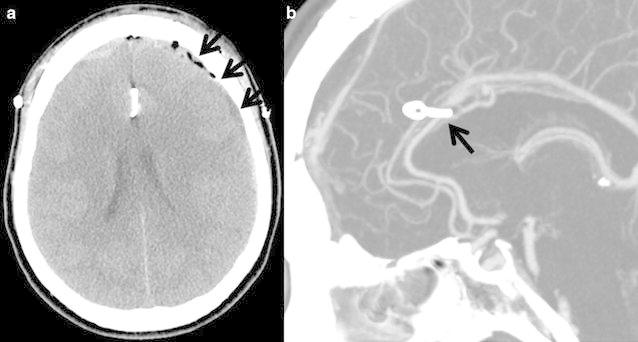
**a**, **b** Postoperative CTA revealed that previous subdural hematoma was evacuated and aneurysm was clipped without a remnant sac (*arrow*)

A 48-years-old female presented to a local hospital complaining of a severe headache and underwent a brain computed-tomography (CT) scan which revealed an acute subdural hematoma. She was transferred to our hospital for further evaluation and treatment. Despite a severe headache, the patient had an alert mental status; more interestingly, the patient also denied a history of trauma. Beside hypertension, which was well-controlled with a combination of a calcium channel blocker and an angiotensin receptor blocker, the patient had no medical or surgical history, and took no other medication, including anticoagulants. Preoperative coagulation tests confirmed the lack of anticoagulant use as they were normal. Brain computed tomography (CTA) and digital subtraction angiography (DSA) were performed to evaluate for vascular abnormalities, and these angiographic studies revealed a 5 × 3 mm sized aneurysm at the A3–A4 junction of left ACA (Fig. 1). On admission to the hospital, to protect against re-bleeding, the aneurysm was clipped through a unilateral interhemispheric approach via a bicoronal craniotomy and the SDH was evacuated. Cerebrospinal fluid was noted to be clear and free of blood products. SAH was not observed at the aneurysm site, with only the rupture point detectable at the site of the aneurysm (Fig. 2). Following the emergency operation, the patient’s treatment continued in the intensive care unit, from which she was transferred to the floor on postoperative day seven. The patient walked unaided out of the hospital 2 weeks after surgery, returning to her normal, daily routine and only complaining of a mild headache. The patient did not have any complications after surgery. Follow up brain CTA revealed that the previous subdural hematoma was evacuated and the aneurysm at the A3–A4 junction was clipped without a remnant sac (Fig. 3). Moreover, the patient’s Glasgow Outcome Scale (GOS) measured five points 9 months after surgery.

## Discussion

The majority of aSDH cases (95 %) are associated with head injuries (Marbacher et al. 2010; O’Leary and Sweeny 1986). A brain CT scan allows bleeding to be distinguished from other causes such as hemorrhagic contusions, skull fractures, and punctuate hemorrhage (Marbacher et al. 2010). Detection of a hemorrhage in a common aneurysm site that presents as SAH can allow for discovering the origin of the bleed (Ohkuma et al. 2003). In the annals of neurosurgery, only a few cases of intracranial aneurysm rupture have been reported to manifest solely as an aSDH without SAH (Nonaka et al. 2000). While we are not aware of specific radiologic features of aneurysmal aSDH reported in the literature (Marbacher et al. 2010), we nonetheless posit that for patients who present with aSDH without a history of head trauma, an aneurysmal rupture should be included in the differential diagnosis.

There are several mechanisms to explain the generation of aSDH after aneurysm rupture: (1) The rupture of the aneurysmal causes small bleeding with adhesions to the arachnoid membrane, and secondary aneurysmal rupture leads to a direct bleed into the subdural space (Kocak et al. 2009). (2) High systolic pressure during aneurysmal rupture directly breaks the arachnoid membrane and generates hematomas in the subdural space (Kocak et al. 2009). (3) Rapid accumulation of blood under pressure from the leaking aneurysm passes into the subarachnoid membrane (Kocak et al. 2009). (4) Acute enlargement of an intra-cavernous aneurysm results in erosion of the cavernous sinus wall (Nozar et al. 2002). Subsequently, rupture of this lesion causes subdural hematoma (Kocak et al. 2009). (5) A distal ACA aneurysm primarily adherent to the falx or the dura bleeds into the subdural space upon rupture (Barton and Tudor 1982; Hatayama et al. 1994; Mansour et al. 2014; Oh et al. 2011).

**Fig. 4:**
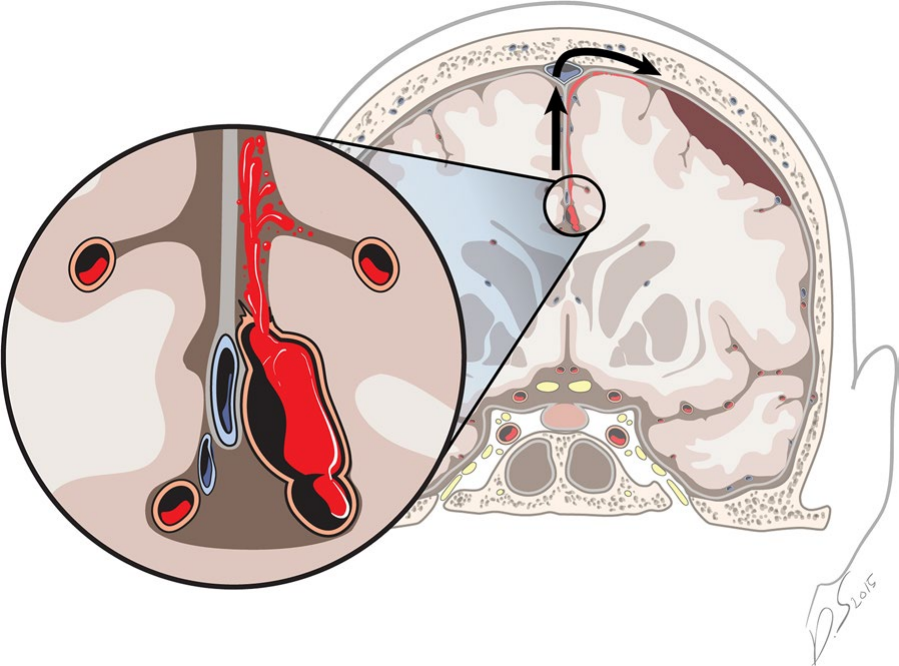
Overview and detailed depiction of the distal anterior cerebral artery aneurysm manifesting as an acute subdural hematoma

In the present case, we hold that the fifth mechanism described above is responsible for the subdural hematoma in our patient. Similar to other reports about ACA aneurysm rupture responsible for aSDH without SAH (Table 1) (Hatayama et al. 1994; Weil et al. 2010), during surgery, the sac of the ruptured ACA aneurysm was glued to the anterior falx with surrounding traces of hemorrhage. A relatively fresh hematoma was observed in the subdural space and was evacuated as much as possible. In rare cases, a ruptured aneurysm adhering tightly to the falx results in pure aSDH without SAH. In addition, pure aSDH without SAH is rarely induced by the rupture of a cortical aneurysm or a giant aneurysm extending into the subdural space (Hori et al. 2005; Nonaka et al. 2000; Yasui et al. 1995) (Fig. 4).

**Table 1:** Summary of the cases of pure aSDH (without SAH) caused by ACA aneurysm rupture

Authors	Age/sex	Signs and symptoms	Site of aSDH	Site of aneurysm	Treatment	Outcome (GOS)
Watanabe et al. (1991)	51, M	Coma	Convexity and interhemispheric	Distal ACA	Evacuation and clipping	D (1)
Ragland et al. (1993)	55, M	Coma	Convexity	AcomA	Evacuation	D (1)
Hatayama et al. (1994)	55, M	Coma	Convexity and interhemispheric	Distal ACA	Evacuation and clipping	GR (5)
Hatayama et al. (1994)	66, F	Coma	Convexity and interhemispheric	Distal ACA	Evacuation and clipping	MD (4)
Katsuno et al. (2003)	63, F	Headache, nausea	Convexity and interhemispheric	Distal ACA	Evacuation and clipping	GR (5)
Gilad et al. (2007)	47, M	Nausea, vomiting	Sella, spinal canal	AcomA	Coiling^a^	GR (5)
Tomaya et al. (2012)	54, M	Headache, nausea	Convexity and tentorium	A1–A2 junction	Evacuation and clipping	GR (5)
Present case 2015	48, F	Headache	Convexity and interhemispheric	Distal ACA	Evacuation and clipping	GR (5)

*AcomA* anterior communicating artery, *ACA* anterior cerebral artery, *aSDH* acute subdural hematoma, *SAH* subarachnoid hemorrhage, *A1–A2* = anterior cerebral artery 1–2 portion, *GOS* Glasgow outcome scale [D = dead (1), *PVS* persistent vegetative state (2), *SD* severe disability (3), *MD* moderate disability (4), *GR* good recovery (5)]

^a^The patient underwent only coiling and was discharged 5 days afterward

From previous reports, the incidence of aneurysmal rupture with aSDH is dependent on the location of the ruptured aneurysm (Cho et al. 2011; Mansour et al. 2014; Marbacher et al. 2010). In cases such as these, O’Sullivan et al. (1994) reported the location to be at the internal carotid artery (ICA) in 43 %, the middle cerebral artery (MCA) in 22 %, the anterior communicating artery (AcomA) in 22 %, the vertebrobasilar artery in 4 %, and in other locations in 9 % of cases. In slight contrast, Ohkuma et al. (2003) found the location to be at the ICA in 38 %, the MCA in 46 %, and A-com/ACA in 15 % of cases, while Strang et al. reported locations at the ICA in 53 %, and the ACA in 27 % of cases (Marbacher et al. 2010).

Because a segment of the ICA courses to the Circle of Willis through the subdural space, ICA aneurysm ruptures have a greater tendency to result in subdural hematomas (SDH) (Marbacher et al. 2010). The perianeurysmal environment associated with ICA aneurysm rupture leads to a specific bleeding pattern. It could be that an aneurysm invading the basilar cisterns makes an aSDH, for example, in ICA or AcomA cases. However, when an aneurysm is surrounded by brain tissue, as in the cases of MCA aneurysms, rupture more likely results in SAH, ICH or IVH without an aSDH (Schuss et al. 2013). It is exceedingly rare for a posterior circulation aneurysm rupture, such as a vertebrobasilar artery aneurysm, to prevent as an aSDH due to Lilliquist’s membrane surrounding the basilar artery: this membrane is thicker than the arachnoid membrane at other parts of the neurovasculature, and as such, seldom allows for bleeding into the subdural space (Biesbroek et al. 2012).

Patients with spontaneous aSDH due to the rupture of aneurysm had worse clinical outcomes than those without aSDH (Biesbroek et al. 2013; Marbacher et al. 2010). Matthjis et al. demonstrated that the rate of poor outcome in patients with aSDH due to aneurysm rupture was 79 % on admission and 75 % at 3 months after discharge, whereas the rate for the non-aSDH group was 50 % on admission and 35 % at 3 months after discharge (Biesbroek et al. 2013). Besides, the group with aSDH due to aneurysm rupture had a higher mortality than the group with only traumatic aSDH (Mansour et al. 2014), with the main reason for deterioration attributed to increased intracranial pressure (Biesbroek et al. 2013). In most cases, preoperative herniation and greater shift in the midline in the setting of large hematomas already existed (Weir et al. 1984). Accordingly, urgent decompression is required to prevent severe deterioration. As a rule, clinical outcomes correlated with the initial clinical and radiological status of each patient; good condition on admission meant a good prognosis at discharge.

The treatment strategy of spontaneous aSDH depends on the patient’s initial condition (Marbacher et al. 2010). Should the patient present with spontaneous aSDH causing life-threatening brain swelling and herniation, urgent evacuation and decompression must be performed first before further evaluations about vascular abnormalities occur (Mansour et al. 2014; Oh et al. 2011; Ohkuma et al. 2003; Schuss et al. 2013), as this emergency surgery prevents a poor outcome (Schuss et al. 2013). After recovery, further evaluation utilizing angiographic studies such as brain CTA or DSA can be considered. Subsequently, aneurysm clipping or coil embolization must be carried out in accordance with the site of the aneurysm. If the condition of patient is stable, an evaluation for vascular abnormalities should be conducted before surgical treatment (Mansour et al. 2014; Oh et al. 2011). DSA is more accurate, but brain CTA has advantages of shorter exam time and relatively good scanning quality (Kocak et al. 2009). After the aneurysm has been observed in brain images, aneurysm clipping and hematoma evaluation proceed. Following surgical treatment, coli embolization may be performed depending on the site of the aneurysm (O’Sullivan et al. 1994). If the SDH is small, coil embolization without hematoma evacuation may be performed (Marbacher et al. 2010). As with some authors, since about 90 % of aneurysmal SAH is generated in the anterior circulation, explorative craniotomy should be considered for patients suffering from a spontaneous aSDH (Weir et al. 1984).

## Conclusion

Spontaneous SDH due to the rupture of an intracranial aneurysm is rare. It is worth nothing, however, that the clinical outcomes of patients who present in this way are poor, thereby necessitating the need for aneurysm rupture to be considered in the differential diagnosis in patients presenting with spontaneous SDH. CTA or DSA may be helpful to detect vascular abnormalities in patients who present with acute subdural hematoma without a history of trauma.
